# One-step Conversion of Levulinic Acid to Succinic Acid Using I_2_/*t*-BuOK System: The Iodoform Reaction Revisited

**DOI:** 10.1038/s41598-017-17116-4

**Published:** 2017-12-21

**Authors:** Ryosuke Kawasumi, Shodai Narita, Kazunori Miyamoto, Ken-ichi Tominaga, Ryo Takita, Masanobu Uchiyama

**Affiliations:** 1 Graduate School of Pharmaceutical Sciences, The University of Tokyo, 7-3-1 Hongo, Bunkyo-ku, Tokyo, 113-0033 Japan; 2 National Institute of Advanced Industrial Science and Technology (AIST), Central 5, 1-1-1 Higashi, Tsukuba, Ibaraki, 305-8565 Japan; 3 Advanced Elements Chemistry Team, RIKEN Center for Sustainable Resource Science, 2-1 Hirosawa, Wako-shi, Saitama, 351-0198 Japan

## Abstract

The iodoform reaction has long been used as a qualitative test for acetyl and/or ethanol units in organic molecules. However, its synthetic applications are quite limited. Here, we describe a tuned iodoform reaction for oxidative demethylation reaction with I_2_ and *t*-BuOK in *t*-BuOH, in which *in situ-*generated *t*-BuOI serves as the chemoselective iodinating agent. This system enables one-step conversion of levulinic acid to succinic acid, a major four-carbon chemical feedstock. This oxidative demethylation is also applicable to other compounds containing an acetyl group/ethanol unit, affording the corresponding carboxylic acids in a selective manner.

## Introduction

**Figure 1:**
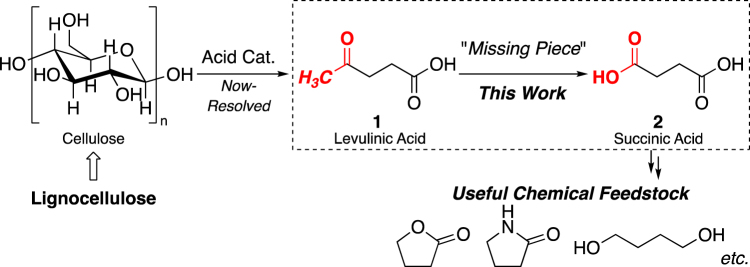
Chemical feedstock production from lignocellulose *via* **1**.

Given the high cost, unsustainability, and environmental burden of petroleum, alternative processes for production of key chemical building blocks from non-petroleum-based resources such as natural gas, coal, or biomass, are of great interest^1–6^. For example, a fermentation route from edible biomass (glucose) to succinic acid **2** has recently been commercialized^7^. But, the use of non-edible lignocellulosic biomass as a source of valuable chemicals would be even more useful on the grounds of low cost and sustainability^8,9^. One of our group has established that simple treatment of lignocellulose with Lewis/Brønsted acid catalyst systems in water or methanol efficiently affords levulinic acid **1** or its methyl ester in a single step^10–15^. Therefore, a direct, simple chemical conversion of **1** to **2** (Fig. 1) is needed, because **2** is an important four-carbon feedstock for conversion to a range of useful chemicals, such as 1,4-butanediol, γ-butyrolactone, and 2-pyrrolidone, as well as being a raw material for bio-based polymers and green sustainable plastics^16–18^.

**Figure 2:**
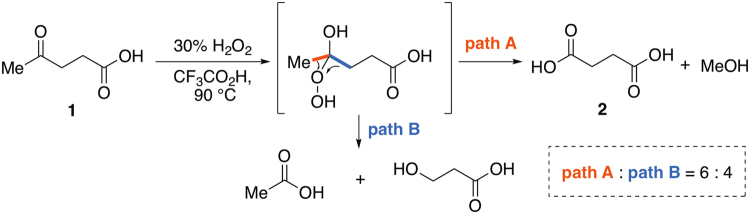
Unusual *terminal* Baeyer-Villiger oxidation using H_2_O_2_/CF_3_CO_2_H.

Thus, a straightforward and practical methodology for conversion of non-edible lignocellulose to succinic acid **2** *via* **1** should have important industrial applications. However, existing chemical methods for the conversion of **1** to **2** involve tedious multi-step synthesis^19–21^, harsh reaction conditions^22,23^, use of toxic heavy metals^24^, and/or low chemical yields^25,26^. For example, the gas-phase oxidation of **1** with vanadium catalyst affords a reasonable yield of **2**, but requires high temperature (375 °C)^22^. Silica-coated magnetic nanoparticle-supported Ru(III) catalyzes the oxidation at somewhat lower temperature (150 °C), but 10 bar pressure of O_2_ is needed^23^. In 2015, a convenient method using aq. 30% H_2_O_2_ in acidic media was reported by Mascal, based on an unusual *terminal* Baeyer-Villiger oxidation (BVO)^27,28^ of **1** to afford **2** in 62% yield (Fig. 2)^29^. However, large amounts of acetic acid and 3-hydroxypropionic acid are formed concomitantly *via* normal BVO (*ca*. 6:4 selectivity). Thus, the development of a kinetically well-controlled transformation from **1** to **2** under mild conditions is still highly desirable. Herein, we report a new protocol for the direct conversion of **1** to **2** at room temperature in high chemical yield. Customization of the haloform reaction has enabled us to achieve one-step, regioselective oxidative demethylation of **1** under mild conditions. The procedure has also been successfully applied to a range of methyl ketones and secondary ethanol derivatives.

## Results and Discussion

### Synthesis of succinic acid from levulinic acid

**Figure 3:**
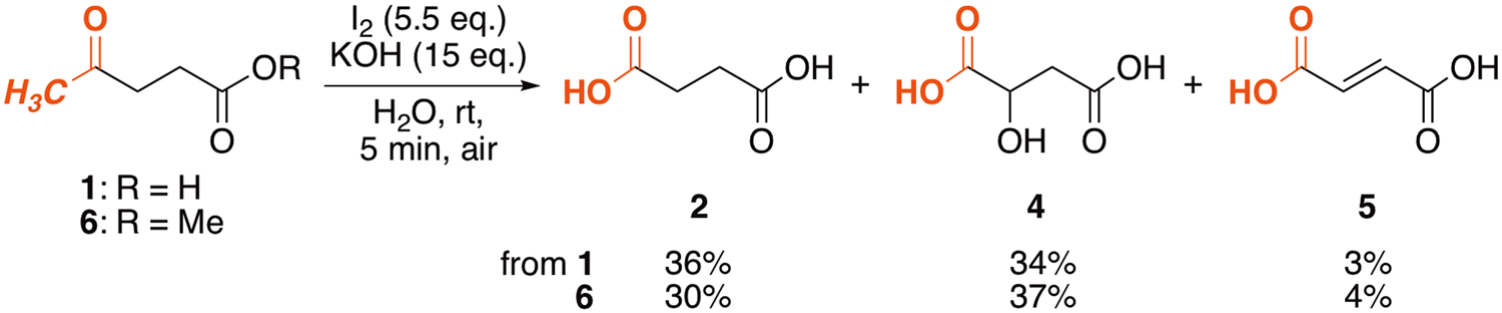
“Classical” iodoform reaction of **1** and **6** to afford succinic acid **2**.

The haloform reaction has traditionally been used as a chemical test to determine the presence of a methyl ketone. However, its synthetic use as for oxidative demethylation of methyl ketones is problematic because of side reactions such as internal α-CH oxidation/halogenation, aldol reaction, Favorskii rearrangement, *etc*. Indeed, only limited success has been reported to date, and the substrate generality and chemoselectivity of this reaction are therefore still unclear^30–34^. We thus commenced our studies with an examination of the “classical” iodoform reaction of **1**. Exposure of **1** to a large excess of I_2_ and KOH in water at room temperature in air resulted in immediate precipitation of canary-yellow iodoform **3** (CHI_3_) and **2** was obtained in 36% yield, but significant side reactions affording 2-hydroxysuccinic acid **4** ^35^ (34%) and fumaric acid **5** (3%) were also observed (Fig. 3). Decreasing the amounts of both reagents (I_2_ and KOH) significantly decreased the yield of **2**, but failed to improve the chemoselectivity. The use of methyl levulinate **6** gave comparable results in terms of reactivity and selectivity. No further oxidation of **2** was observed under the reaction conditions, indicating that the **4** and **5** should be produced directly from starting material **1** or **6** (*i.e*., not *via* **2**).

**Figure 4:**
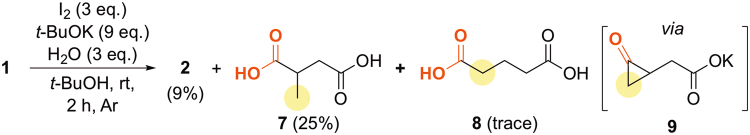
Unsuccessful result of one-shot addition of reagents without “pre-treatment” for *in situ* generation of *t*-BuOI.

After extensive experimentation to find a better base/solvent system than HO^–^/water, we found that the combination of *t-*BuO^–^ (base) and *t-*BuOH (solvent) improved the chemo/regioselectivity of the oxidative demethylation reaction (Table 1). This reaction system has a number of attractive features compared to the prototype iodoform conditions, as follows. Firstly, the *t-*BuOH (tertiary alcohol) is inherently resistant to the oxidation conditions, which represents an obvious advantage over other common alcohols, such as MeOH, EtOH, *i*-PrOH, *etc* ^36^. The use of *t*-amyl alcohol gave comparable results. Secondly, *t*-BuO^–^ base would abstract a terminal α-methyl proton with kinetic preference over an internal α-proton. Thirdly, *t-*BuOI^37–40^ would be generated *in situ*, serving as the chemoselective iodinating agent^41^. These three factors would result in high selectivity, so that the internal CH_2_ group remains almost intact under these conditions. The reaction protocol in Table 1 involves i) pre-treatment with 3 equivalents (theoretical amount) of iodine and theoretical amount of *t-*BuOK in *t-*BuOH in order to form *t-*BuOI, followed by ii) addition of H_2_O, and then iii) a solution of **1** in *t*-BuOH, affording the desired product **2** with high selectivity. It is important to note that the pre-treatment is crucial for selective formation of **2**. Direct addition of I_2_ to the mixture of **1** and *t*-BuOK in *t*-BuOH was unsatisfactory, resulting in a low yield (9%) of **2** and low selectivity: 2-methylsuccinic acid **7** (25%) and trace amount of glutaric acid **8** were formed, probably through Favorskii-type rearrangement *via* cyclopropanone intermediate **9** (Fig. 4)^42^.

**Table 1:** Procedure and optimization of iodoform reaction of **1** with *in situ-*generated *t*-BuOI^a^.


Entry	**1** in *t*-BuOH	H_2_O	Yield (%)^*c*^	
	(M)^*b*^	(eq.)	**2**	**5**
1	1.0	1.0	58	2
2	1.0	3.0	63	2
3	1.0	10	55	2
4^*d*^	1.0	3.0	69	2
5^*d*^	0.2	3.0	87(83)	2(2)
6^*d*^	2.2	3.0	64	2
7^*d,e*^	0.2	3.0	(67)	2
8^*d,f*^	0.2	3.0	8	4
9^*d*^	0.2	5.0	86	3
10^*d*^	0.2	1.0	85	2

^*a*^Reaction conditions: I_2_ (3 eq.), *t*-BuOK (9 eq.) in *t*-BuOH at rt for 5 min under argon. Initial net concentration of **1** in *t*-BuOH is 0.05 M.

^*b*^Concentration of stock solution of **1** in *t*-BuOH.

^*c*1^H NMR yields. Numbers in parentheses are isolated yields.

^*d*^ **1** in *t*-BuOH was added dropwise to a *t*-BuOI solution in *t*-BuOH over 10 min.

^*e*^10 mmol scale.

^*f*^ *t*-BuONa was used instead of *t*-BuOK.

We found that the amount of water and the concentrations of the reagents are critical factors affecting the reaction efficiency. Increased chemical yields of **2** were obtained by the use of 1–10 equivalents of water (entries 1–3). Slow addition (~10 min) of **1** to the solution of *t*-BuOH improved the reaction outcome (entries 4–10). The best result was obtained when 0.2 M of **1** in *t*-BuOH was used as a stock solution (entry 5), while 2.2 M solution of **1** gave a lower yield of **2** (entry 6). The optimized conditions could be scaled-up to 10 mmol (1.16 g) without any column purifications, although slight decrease in efficiency was observed (entry 7). It should be noted that the counter-cation of the base and the source of the halogen also played critical roles in determining the yield of this oxidative demethylation reaction. The use of *t*-BuONa instead of *t*-BuOK dramatically decreased the yield of **2** (entry 8), probably due to the relatively poor solubility of *t*-BuONa. We also found that other halogen sources, such as *t*-BuOCl and *t*-BuOBr, were ineffective, yielding only a small amount of **2** (for details, see Supporting Information, Figure S1).

### Synthesis of succinic acid from cellulose

**Figure 5:**
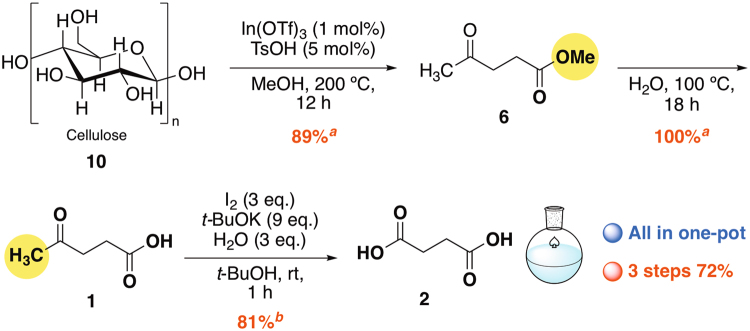
Direct one-pot three steps synthesis of **2** from cellulose **10**. ^*a*1^H NMR yields. ^*b*^Isolated yield after dehydration of **2**.

To elucidate the efficiency of modified iodoform reaction, direct one-pot synthesis of succinic acid **2** from cellulose **10** was investigated (Fig. 5). In(OTf)_3_–TsOH catalyzed refining of **10** yielding methyl levulinate **6**, proceeded in high yield^11,12^. The reaction mixture was then hydrolyzed in water by remaining acids to give **1** quantitatively, which was followed by the demethylated under optimized reaction conditions to afford **2** in 81% yield (72%, three steps).

### Scope and limitations

**Figure 6:**
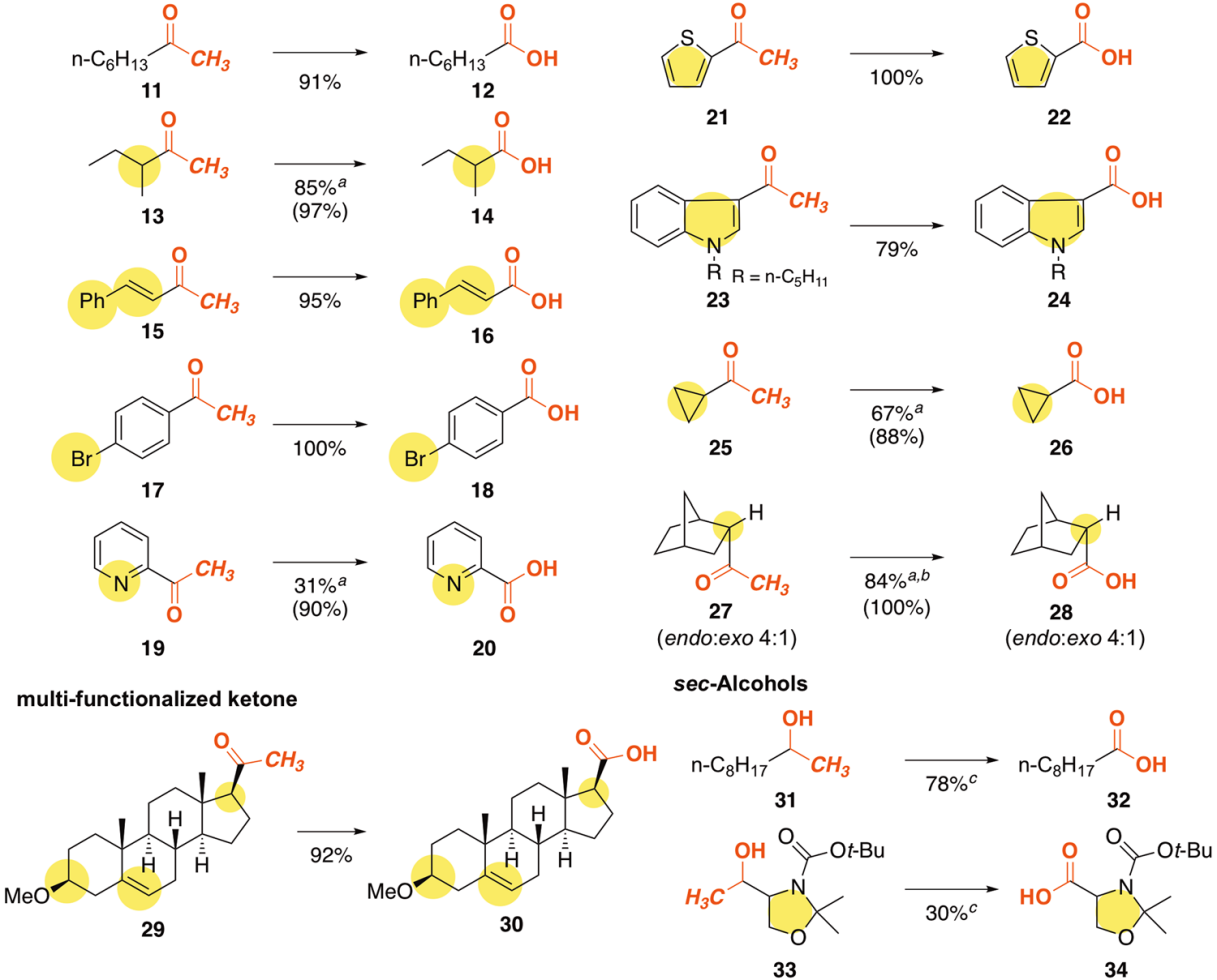
Oxidative demethylation of ketones and alcohols. The reactions were performed after pre-treatment of I_2_ (3 eq.) and *t*-BuOK (9 eq.), using 3 eq. of H_2_O at room temperature for 1 h (same as the conditions in Table 1, entry 5). Isolated yields. (^1^H NMR yields are shown in parentheses). ^*a*^Isolated yields after benzylation. ^*b*^The *endo*:*exo* ratio was unchanged during the course of reaction, as determined by ^1^H NMR measurements. ^*c*^ *t*-BuOK (10 eq.) and I_2_ (4 eq.) were used.

These reaction conditions were also applicable to various methyl ketones and secondary ethanol derivatives (Fig. 6). Simple methyl ketones such as 2-octanone **11** and *sec*-butyl methyl ketone **13** smoothly underwent oxidative demethylation reactions yielding corresponding carboxylic acids **12** and **14** in high yields, respectively. 4-Phenylbut-3-en-2-one **15** was efficiently converted to the corresponding carboxylic acid **16** in 95% yield. *t*-BuOI-mediated conditions were found to be suitable for a wide range of aromatic- and heteroaromatic systems. Not only electron deficient (**17** and **19**) but also electron rich (**21** and **23**) aryl methyl ketones serve as good substrates. In classical haloform reaction, electron rich aryl groups are troublesome substrates, but they were available in our system^31,34^. For these heterocycles, neither iodination of aromatic ring nor decarboxylation of products (**22** and **24**) were observed. Cyclopropyl methyl ketone **25** gave the desired product **26** in high yield and the cyclopropane ring remained intact. The stereochemistry of the starting materials (**27** and **29**) was almost completely retained in the products (**28** and **30**)^43^, implying high regioselectivity of the iodination step with these substrates. This system was also applicable to a secondary ethanol derivative **31**, affording nonanoic acid **32** in 71% yield *via* oxidation/demethylation sequences. Similarly, *N*-acyl-*N*,*O*-acetal **33** undergoes demethylation **34** selectively, albeit in a moderate yield.

## Conclusions

In summary, we have developed a simple, chemo-selective, cost-effective oxidative demethylation reaction of methyl ketones utilizing *in situ*-generated *t*-BuOI, which enables one-pot conversion of levulinic acid **1** to succinic acid **2** at room temperature. **2** is an important chemical feedstock, and our study offers the efficient chemical process to provide **2** from non-edible lignocellulose *via* **1**. This system was also shown to be applicable to various substrates containing acetyl/ethanol units. Further studies to expand the scope of the reaction and to elucidation of the reaction mechanism with the help of theoretical and spectroscopic studies are in progress in our laboratory.

## Method

### General Information

IR spectra were recorded on a JASCO FT-IR 4700 spectrometer. ^1^H NMR and ^13^C NMR spectra were obtained on a Bruker AVANCE III HD spectrometer. Chemical shifts (δ) are reported in parts per million (ppm) downfield from internal Me_4_Si. Mass spectra (MS) were obtained on a Bruker micrOTOF-QIII spectrometer or an Agilent Model 5977B spectrometer. Preparative thin-layer chromatography (TLC) was carried out on pre-coated plates of silica gel (MERCK, silica gel F-254).

### Substrate

3-Acetyl-1-pentyl-1*H*-indole (**23**) was prepared from commercially available 3-acetylindole in 2 steps according to the literature procedure^44,45^. A 4:1 mixture of *endo-* and *exo-*2-acetylnorbornane (**27**) was prepared from commercially available *endo-* and *exo-*2-acetylnorbornan-5-ene by hydrogenation with Pd/C and H_2_ according to the literature procedure:^46^ 3-β-Methoxy-5-pregnen-20-one (**29**) was prepared from commercially available 3-β-hydroxy-5-pregnen-20-one according to the literature procedure^47^. *N*-(*tert*-Butoxycarbonyl)-2,2-dimethyl-4-(1-hydroxyethyl)oxazolidine (**33**) was prepared from corresponding aldehyde according to the literature procedure^48^.

### Oxidative demethylation of levulinic acid (1) in water

To a stirred solution of KOH (315 mg, 5.6 mmol) and levulinic acid (**1**) (53 mg, 0.40 mmol) in water (10 mL) was added I_2_ (560 mg, 2.2 mmol) and the resulting yellow suspension was stirred at room temperature for 5 min. After treatment of HCl-acidified reaction mixture (pH *ca*. 1) with excess (≥1 mL) 30% aqueous H_2_O_2_, the mixture was washed several times with dichloromethane until the color of I_2_ and CHI_3_ faded. The aqueous phase was then concentrated *in vacuo* and extracted with acetone several times, which was concentrated in an aspiratory vacuum to give a mixture of dicarboxylic acids as a white powder. ^1^H NMR analysis (1,1,2,2-tetrachloroethane as an internal standard) showed the formation of succinic acid (**2**) (36%), 2-hydroxysuccinic acid (**4**) (34%), and fumaric acid (**5**) (4%) (Fig. 3).

#### Succinic acid (**2**)

colorless needles (recrystallized from acetone): IR (neat): ν = 3364–2159, 1680, 1410, 1306, 1196, 892, 800, 635, 581, 545 cm^−1^; ^1^H NMR (500 MHz, D_2_O): δ = 2.80 ppm (s, 4 H); ^13^C NMR (125 MHz, D_2_O): δ = 177.0, 28.7 ppm; MS (ESI (–)): *m/z*: 117 [(M-H)^–^]^49^.

### Demethylation of levulinic acid (1) with I_2_ and *t*-BuOK in *t*-BuOH

To a stirred solution of *t*-BuOK (95 mg, 0.85 mmol) in distilled *t*-BuOH (1.4 mL) was added I_2_ (72 mg, 0.28 mmol) and the mixture was stirred at room temperature for a few minutes. After fading the color of I_2_, the beige suspension was added H_2_O (5.0 mg, 0.28 mmol) and then the solution of levulinic acid (**1**) (11 mg, 0.092 mmol) in dry *t*-BuOH (0.48 mL) dropwise during 10 min. After the reaction mixture was stirred at room temperature for additional 1 h, the mixture was concentrated *in vacuo* and dissolved in water. After treatment of HCl-acidified reaction mixture (pH *ca*. 1) with excess ( ≥ 1 mL) 30% aqueous H_2_O_2_, the mixture was washed several times with dichloromethane until the color of I_2_ and CHI_3_ faded. The aqueous phase was then concentrated *in vacuo*, and extracted with acetone several times, which was followed by the concentration in an aspiratory vacuum to give the mixture of dicarboxylic acids (10 mg) as a white powder. ^1^H NMR analysis (1,4-dioxane as an internal standard) showed the formation succinic acid (**2**) (83%), fumaric acid (**5**) (2%), and 2-hydroxysuccinic acid (**4**) (2%). Further recrystallization with acetone gave pure succinic acid (**2**) as colorless needles (9 mg, 83%) (Table 1, entry 5).

### One-pot synthesis of succinic acid from cellulose

1^st^ step (*Caution!* the reaction should be carried out behind the safety screen): According to the literature procedure^11^, cellulose (**10**) (428 mg, 2.64 mmol), indium(III) trifluoromethanesulfonate (22.4 mg 0.04 mmol), and *p*-toluenesulfonic acid (38 mg, 0.2 mmol) were suspended in methanol (20 mL) in a Schlenk flask under argon and vigorously stirred at 200 °C for 12 h, the reaction mixture was cooled to room temperature and concentrated under an aspiratory vacuum to give brown oil. ^1^H NMR analysis (1,4-dioxane as an internal standard) showed the formation of methyl levulinate (**11**) (89%), fumaric acid (**5**) (2%), and 2-hydroxysuccinic acid (**4**) (2%). Further recrystallization with acetone gave pure succinic acid (**2**) as colorless needles (Fig. 5).

2^nd^ step: To the mixture was added H_2_O (10 mL) and stirred at 100 °C for 18 h until the disappearance of **11**. After the mixture was cooled to room temperature, the mixture was concentrated *in vacuo* to give brown oil. ^1^H NMR analysis (1,4-dioxane as an internal standard) showed the formation of levulinic acid (**1**) (100%). The residue was dissolved in *t*-BuOH (10 mL) and transferred into a syringe in order to use for further transformations.

3^rd^ step: To a stirred solution of *t*-BuOK (2.4 g, 21.2 mmol) in distilled *t*-BuOH (30 mL) in a Schlenk flask was added I_2_ (1.8 g, 7.05 mmol) and the mixture was stirred at room temperature for a few minutes. After fading the color of I_2_, the beige suspension was added H_2_O (127 mg, 7.05 mmol) and then the above solution of levulinic acid (**1**) (2.35 mmol) in dry *t*-BuOH (10 mL) dropwise during 10 min. After the reaction mixture was stirred at room temperature for additional 1 h, the mixture was concentrated *in vacuo* and dissolved in water. After treatment of HCl-acidified reaction mixture (pH *ca*. 1) with excess ( ≥ 2 mL) 30% aqueous H_2_O_2_, the mixture was washed several times with dichloromethane until the color of I_2_ and CHI_3_ faded. The aqueous phase was then concentrated *in vacuo*, and extracted with acetone several times. After the addition of acetic anhydride, the mixture was heated at 80 °C for 8 h. The mixture was cooled to room temperature and concentrated *in vacuo* to give succinic anhydride as a white powder (190 mg, 81%).

#### Succinic anhydride

^1^H NMR (500 MHz, DMSO-*d*_6_): δ = 2.91 ppm (s, 4 H)^50^.

### General procedure for demethylation of methyl ketones with I_2_ and *t*-BuOK in *t*-BuOH. A typical example: demethylation of 2-octanone (**11**)

To a stirred solution of *t*-BuOK (85 mg, 0.75 mmol) in distilled *t*-BuOH (1.4 mL) was added I_2_ (72 mg, 0.28 mmol) and the mixture was stirred at room temperature for a few minutes. After fading the color of I_2_, the beige suspension was added H_2_O (5.0 mg, 0.28 mmol) followed by the solution of 2-octanone (**11**) (12 mg, 0.093 mmol) in dry *t*-BuOH (0.46 mL) dropwise during 10 min. After the reaction mixture was stirred at room temperature for additional 1 h, the mixture was concentrated *in vacuo*. The residue was dissolved in water and washed with dichloromethane three times. HCl-acidified aqueous phase was extracted with dichloromethane two times and then with diethyl ether. The combined organic phase was washed with aqueous Na_2_S_2_O_3_ solution and brine, dried over Na_2_SO_4_, filtered, and concentrated under an aspiratory vacuum to give heptanoic acid (**12**) (12 mg) as an oil (93% purity, confirmed by ^1^H NMR). The residue was dissolved in DMF (2.0 mL) and added K_2_CO_3_ (13.8 mg, 0.1 mmol), benzyl bromide (18 mg. 0.1 mmol), and 18-crown-6 (8.0 mg, 0.030 mmol). After heating the solution at 85 °C for 24 h, the mixture was concentrated *in vacuo* and purified by silica gel column chromatography (hexane:toluene = 1:1) to give benzyl heptanoate (7.2 mg) as a pale yellow oil:^51^ IR (neat): ν = 2958, 2928, 2857, 1737, 1455, 1376, 1216, 1160, 1102, 1003, 733, 696, 527 cm^−1^; ^1^H NMR (500 MHz, CDCl_3_): δ = 7.40–7.30 (m, 5 H), 5.11 (s, 2 H), 2.35 (t, *J* = 7.5 Hz, 2 H), 1.64 (quint, *J* = 7.5 Hz, 2 H), 1.36–1.24 (m, 6 H), 0.87 ppm (t, *J* = 7.0 Hz, 3 H); ^13^C NMR (125 MHz, CDCl_3_): δ = 173.7, 136.2, 128.5, 128.17 (*o*, *p*), 66.1, 34.4, 31.4, 28.8, 24.9, 22.5, 14.0 ppm. (Fig. 6)

Products **17**, **19**, **23**, **25**, and **31** were obtained by recrystallization of residue from hexane.

### General procedure for demethylation of ketone with I_2_ and *t*-BuOK in *t*-BuOH (in case of volatile products). A typical example: demethylation of acetylcyclopropane (25)

To a stirred solution of *t*-BuOK (81 mg, 0.72 mmol) in distilled *t*-BuOH (1.4 mL) was added I_2_ (69 mg, 0.27 mmol) and the mixture was stirred at room temperature for a few minutes. After fading the color of I_2_, the beige suspension was added H_2_O (4.8 mg, 0.27 mmol) and then the solution of acetylcyclopropane (**25**) (7.5 mg, 0.089 mmol) in dry *t*-BuOH (0.43 mL) dropwise during 10 min. After the reaction mixture was stirred at room temperature for additional 1 h, the mixture was concentrated *in vacuo*. The residue was suspended in MeCN (1.8 mL) and added benzyl bromide (17 mg, 0.097 mmol) and 18-crown-6 (3.0 mg, 0.012 mmol). After heating the solution at 75 °C for 24 h, the mixture was concentrated in an aspiratory vacuum to give an oil, which was purified by silica gel column chromatography (hexane then hexane-ethyl acetate = 1:1) to give benzyl cyclopropanecarboxylate as an oil. ^1^H NMR analysis (1,4-dioxane as an internal standard) showed the formation of benzyl cyclopropanecarboxylate (88%). Further purification by silica gel column chromatography (hexane:toluene = 1:1) to give benzyl cyclopropanecarboxylate (11 mg, 67%) as a pale yellow oil:^52^ IR (neat): ν = 3102–2750, 1725, 1455, 1397, 1360, 1265, 1164, 1065, 1029, 890, 747, 697 cm^−1^; ^1^H NMR (500 MHz, CDCl_3_): δ = 7.40–7.31 (m, 5 H), 5.12 (s, 2 H), 1.66 (tt, *J* = 7.5, 4.5 Hz, 1 H), 1.03 (dt, *J* = 7.5, 4.5 Hz, 2 H), 0.87 ppm (td, *J* = 7.5, 4.5 Hz, 2 H); ^13^C NMR (125 MHz, CDCl_3_): δ = 174.8, 136.2, 128.6, 128.2 (3 C), 66.3, 12.9, 8.6 ppm; MS: *m/z* (%): 176 (30) (*M* ^+^), 104 (18), 91 (100), 77 (32), 69 (56), 65 (31), 51 (16) (Fig. 6).

### Tandem oxidation–demethylation of 2-decanol (**31**) with I_2_ and *t*-BuOK in *t*-BuOH

To a stirred solution of *t*-BuOK (106 mg, 0.94 mmol) in distilled *t*-BuOH (1.4 mL) was added I_2_ (96 mg, 0.38 mmol) and the mixture was stirred at room temperature for a few minutes. After fading the color of I_2_, the beige suspension was added H_2_O (5.0 mg, 0.28 mmol) and then the solution of 2-decanol (**31**) (15 mg, 0.093 mmol) in dry *t*-BuOH (0.46 mL) dropwise during 10 min. After the reaction mixture was stirred at room temperature for additional 3 h, the mixture was concentrated *in vacuo*. The residue was dissolved in water and washed with dichloromethane three times. HCl–acidified aqueous phase was treated with excess Na_2_S_2_O_3_, and extracted with dichloromethane two times and then with diethyl ether. The combined organic phase was washed with brine, dried over Na_2_SO_4_, filtered, and concentrated under an aspiratory vacuum to give nonanoic acid (**32**) (16 mg) as an oil (71% purity, confirmed by ^1^H NMR). Further purification by silica gel column chromatography (hexane:toluene = 1:1) after benzylation using above-mentioned procedure for **12** gave pure benzyl nonanoate (1.5 mg) as a pale yellow oil^53^: IR (neat): ν = 2954, 2925, 2855, 1736, 1456, 1156, 1108, 734, 696 cm^−1^; ^1^H NMR (500 MHz, CDCl_3_) δ 7.40–7.30 (m, 5 H), 5.11 (s, 2 H), 2.35 (t, *J* = 7.5 Hz, 2 H), 1.64 (quint, *J* = 7.5 Hz, 2 H), 1.35–1.20 (m, 10 H), 0.87 ppm (t, *J* = 7.5 Hz, 3 H); ^13^C NMR (125 MHz, CDCl_3_): δ = 173.7, 136.2, 128.5, 128.2 (3 C), 66.1, 34.4, 31.8, 29.2, 29.14, 29.12, 25.0, 22.6, 14.1 ppm; MS: *m/z* (%): 248 (2) (*M* ^+^), 108 (33), 91 (100), 77 (10), 65 (15) (Fig. 6).

## Electronic supplementary material

Supporting information
