# Supplementary material for: One-step Conversion of Levulinic Acid to Succinic Acid Using I2/t-BuOK System: The Iodoform Reaction Revisited
Source: Sci Rep. 2017 Dec 21;7:17967. doi: 10.1038/s41598-017-17116-4 (PMC5740180; doi:10.1038/s41598-017-17116-4)
Supplement: Supplementary file 1 — Supporting information [file 41598_2017_17116_MOESM1_ESM.pdf]

SUPPORTING INFORMATION

One-step Conversion of Levulinic Acid to Succinic Acid Using I_2/*t*-BuOK System: The Iodoform Reaction Revisited

Ryosuke Kawasumi,[1] Shodai Narita,[1] Kazunori Miyamoto,[1,*] Ken-ichi Tominaga,[2] Ryo Takita,[3] and Masanobu Uchiyama[1,3,*]

[1] Graduate School of Pharmaceutical Sciences, The University of Tokyo, 7-3-1 Hongo, Bunkyo-ku, Tokyo 113-0033, Japan.
[2] National Institute of Advanced Industrial Science and Technology (AIST) Central 5, 1-1-1 Higashi, Tsukuba, Ibaraki 305-8565, Japan.
[3] Advanced Elements Chemistry Team, RIKEN Center for Sustainable Resource Science, 2-1 Hirosawa, Wako-shi, Saitama 351-0198, Japan.

E-mail: kmiya@ mol.f.u-tokyo.ac.jp (K.M.); uchiyama@mol.f.u-tokyo.ac.jp (M.U.)

Table of Contents

1.	Figure S1	S2
2.	Experiment Details	S3–S5
3.	NMR spectra	S6–S8
4.	Reference	S9

1. Attempted demethylation of levulinic acid with *t*-BuOCl and *t*-BuOBr (Figure S1)

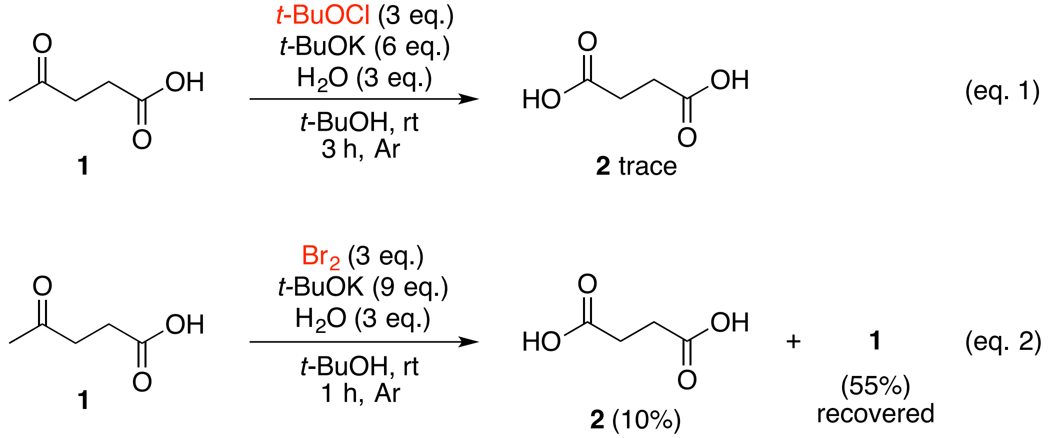

Figure S1. Attempted demethylation of **1** with commercially available *t*-BuOCl (eq. 1) and *in situ* generated *t*-BuOBr (eq. 2). Both of these approaches gave only a small amount of **2**, accompanied by the formation of α-poly-halogenated products (not shown).

2. Experimental details

Oxidative demethylation of methyl levulinate (6) in water (Figure 3). To a stirred solution of KOH (512 mg, 9.1 mmol) and methyl levulinate (**6**) (85 mg, 0.65 mmol) in water (16 mL) was added I_2 (910 mg, 3.6 mmol) and the resulting yellow suspension was stirred at room temperature for 5 min. After treatment of HCl-acidified reaction mixture (pH *ca.* 1) with excess (≥ 1 mL) 30% aqueous H_2O_2, the mixture was extracted several times with dichloromethane until the color of I_2 and CHI_3 faded. The aqueous phase was then concentrated *in vacuo*, and extracted with acetone several times, which was followed by the concentration in an aspiratory vacuum to give the mixture of dicarboxylic acids as a white powder. ^{1}H NMR analysis (1,1,2,2-tetrachloroethane as an internal standard) showed the formation of succinic acid (**2**) (30%), 2-hydroxysuccinic acid (**4**) (37%), and fumaric acid (5) (4%).

Demethylation of levulinic acid (1) with I_2 and *t*-BuOK in *t*-BuOH: an unsuccessful result (Figure 4). To a stirred solution of *t*-BuOK (296 mg, 2.6 mmol) and levulinic acid (**1**) (33 mg, 0.29 mmol) in *t*-BuOH (1.4 mL) was added I_2 (225 mg, 0.89 mmol) and the mixture was stirred at room temperature for 10 min. Then, H_2O (16 mg, 0.86 mmol) was added and stirred for additional 2 h at the temperature. The reaction mixture was evaporated, acidified with aqueous HCl, and concentrated *in vacuo*. ^{1}H NMR analysis (1,4-dioxane as an internal standard) showed the formation of succinic acid (**2**) (9%) and 2-methylsuccinic acid (**7**) (25%).

Demethylation of levulinic acid (1) with Br_2 and *t*-BuOK in *t*-BuOH: an unsuccessful result (Figure S1). To a stirred solution of *t*-BuOK (95 mg, 0.85 mmol) in *t*-BuOH (1.4 mL) was added Br_2 (45 mg, 0.28 mmol) and the mixture was stirred at room temperature for 1 minute. After fading the color of Br_2, the beige suspension was then added H_2O (5.0 mg, 0.28 mmol) and the solution of levulinic acid (**1**) (11 mg, 0.093 mmol) in dry *t*-BuOH (0.47 mL) dropwise during 10 min. After the reaction mixture was stirred at room temperature for additional 1 h, the mixture was concentrated *in vacuo.* ^{1}H NMR analysis (1,4-dioxane as an internal standard) showed the formation of succinic acid (**2**) (10%).

2-Hydroxysuccinic acid (4):[S1] ^{1}H NMR (500 MHz, D_2O): δ = 4.63 (dd, J = 7.0, 5.0 Hz, 1H), 2.95 (dd, J = 16.5, 5.0 Hz, 1H), 2.88 ppm (dd, J = 16.5, 7.0 Hz, 1H); MS (ESI (−)): *m/z*: 133 [$(M–H)^-$].

Fumaric Acid (5):[S2] ^{1}H NMR (500 MHz, D_2O): δ = 6.87 ppm (s, 2H); MS (ESI (−)): *m/z*: 115 [$(M–H)^-$].

2-Methylsuccinic acid (7):[S3] ^{1}H NMR (500 MHz, acetone-d_6): δ = 2.83 (dqd, J = 8.5, 7.0, 6.0 Hz, 1H), 2.69 (dd, J = 17.0, 8.5 Hz, 1H), 2.40 (dd, J = 17.0, 6.0 Hz, 1H), 1.21 (d, J = 7.0 Hz, 3H); MS (ESI (–)): *m/z*: 131 [$(M–H)^-$].

Benzyl 2-methylbutanoate:[S4] a pale yellow oil: IR (neat): ν = 2968, 1732, 1456, 1383, 1177, 1145, 1077, 1012, 748, 696 cm^{-1}; ^{1}H NMR (500 MHz, $CDCl_3$): δ = 7.38-7.29 (m, 5H), 5.12 (s, 2H), 2.43 (tq, *J* = 7.0, 7.0 Hz, 1H), 1.71 (ddq, *J* = 13.5, 7.0, 7.0 Hz, 1H), 1.49 (ddq, *J* = 13.5, 7.0, 7.0 Hz, 1H), 1.16 (d, *J* = 7.0 Hz, 3H), 0.90 ppm (t, *J* = 7.0 Hz, 3H); ^{13}C NMR (125 MHz, $CDCl_3$): δ = 176.6, 136.3, 128.5, 128.1, 128.0, 66.0, 41.1, 26.8, 16.6, 11.6 ppm; MS: *m/z* (%): 192 (12) (M^+), 108 (10), 91 (100), 77 (12), 65 (16), 57 (18).

Cinnamic acid (16):[S5] colorless needles: IR (neat): ν = 3398-2068, 1673, 1628, 1495, 1449, 1419, 1312, 1284, 1221, 978, 942, 767, 707, 682, 590, 543 cm^{-1}; ^{1}H NMR (500 MHz, $CDCl_3$): δ = 7.79 (d, *J* = 16.0 Hz, 1H), 7.56 (m, 2H), 7.41 (m, 3H), 6.46 ppm (d, *J* = 16.0 Hz, 1H); ^{13}C NMR (125 MHz, $CDCl_3$): d = 172.4, 147.1, 134.1, 130.8, 129.0, 128.4, 117.3 ppm; MS: *m/z* (%): 148 (67) (M^+), 147 (100), 131 (19), 103 (31), 91 (20), 77 (26), 51 (15).

4-Bromobenzoic acid (18):[S6] colorless needles: IR (neat): ν = 3325-2175, 1675, 1584, 1421, 1176, 1126, 1066, 1011, 927, 849, 805, 756, 680 cm^{-1}; ^{1}H NMR (500 MHz, $CDCl_3$): δ = 7.83 (d, *J* = 8.0 Hz, 2H), 7.58 ppm (d, *J* = 8.0 Hz, 2H); ^{13}C NMR (125 MHz, $CDCl_3$): δ = 166.0, 131.7, 131.4, 129.8, 127.2 ppm; MS: *m/z* (%): 202 (93), 200 (100) (M^+), 185 (78), 183 (77), 157 (39), 155 (40), 75 (42), 50 (48).

2-Thiophenecarboxylic acid (22):[S7] pale yellow needles: IR (neat): ν = 3341-2227, 1664, 1527, 1430, 1353, 1281, 1106, 1043, 910, 856, 721, 648 cm^{-1}; ^{1}H NMR (500 MHz, $CDCl_3$): δ = 7.90 (dd, *J* = 4.0, 1.5 Hz, 1H), 7.65 (dd, *J* = 5.0, 1.5 Hz, 1H), 7.15 ppm (dd, *J* = 5.0, 4.0 Hz, 1H); ^{13}C NMR (125 MHz, $CDCl_3$): δ = 167.3, 135.0, 134.0, 132.8, 128.1 ppm; MS: *m/z* (%): 128 (91) (M^+), 111 (100), 57 (19).

1-Pentyl-1*H*-indole-3-carboxylic acid (24):[S8] pale pink needles: IR (neat): ν = 3325-2150, 1650, 1525, 1274, 1205, 930, 732 cm^{-1}; ^{1}H NMR (500 MHz, $CDCl_3$): δ = 8.24-8.22 (m, 1H), 7.92 (s, 1H), 7.41-7.32 (m, 1H), 7.32-7.28 (m, 2H), 4.16 (t, *J* = 7.0 Hz, 2H), 1.90 (quint, *J* = 7.0 Hz, 2H), 1.40-1.28 (m, 4H), 0.90 ppm (t, *J* = 7.0 Hz, 3H); ^{13}C NMR (125 MHz, $CDCl_3$): d = 169.7, 136.7, 135.5, 127.0, 122.8, 122.1, 121.9, 110.1, 106.1, 47.2, 29.5, 29.0, 22.3, 13.4 ppm; MS: *m/z* (%): 231 (85) (M^+), 175 (45), 174 (100), 130 (40).

A 4:1 mixture of benzyl 2-*endo*-norbornanecarboxylate and benzyl 2-*exo*-norbornanecarboxylate:[S9] a colorless oil: IR (neat): ν = 2954, 2872, 1731, 1455, 1310, 1164, 1118, 1026, 747, 696 cm^{-1}; ^{1}H NMR (500 MHz, $CDCl_3$): δ = 7.40-7.31 (m, 5H for both isomers), 5.14 (s, 2H for ***endo***), 5.12 (s, 2H for ***exo***), 2.81 (m, 1H for ***endo***), 2.57 (m, 1H for ***endo***), 2.52 (m, 1H for ***exo***), 2.38 (dd, *J* = 8.0, 5.0 Hz, 1H for ***exo***), 2.30 (m, 1H for ***exo***), 2.26 (m, 1H for ***endo***), 1.87 (m, 1H for ***exo***), 1.68 (m, 1H for ***endo***), 1.66 (m, 1H for ***endo***), 1.63-1.18 ppm (m, 6H for ***endo*** and 7H for ***exo***); ^{13}C NMR (125 MHz, $CDCl_3$): δ = 175.9 for ***exo***, 175.0 for ***endo***, 136.4 for both isomers, 128.5 for both isomers, 128.1 for both isomers, 66.1 for ***exo***, 66.0 for ***endo***, 46.5 for ***exo***,46.1 for ***endo***, 41.0 for ***exo***, 40.5 for ***endo***, 40.2 for ***endo***, 37.0 for ***endo***, 36.5 for ***exo***, 36.0 for ***exo***, 34.2 for ***exo***, 31.9 for ***endo***, 29.5 for ***exo***, 29.1 for ***endo***, 28.6 for ***exo***, 24.9 ppm for ***endo***; MS: *m/z* (%): 230 (5) (M^+),

139 (18), 95 (27), 91 (100), 77 (16), 67 (27), 65 (23).

3β-Methoxy-5-androsten-17b-carboxylic acid (30):[S10] a pale yellow powder: IR (neat): ν = 3364-2423, 2933, 1694, 1452, 1381, 1241, 1191, 1099, 935, 798, 728, 698, 523 cm^{-1}; ^{1}H NMR (500 MHz, $CDCl_3$): δ = 5.36 (m, 1H), 3.36 (s, 3H), 3.07 (tt, *J* = 11.5, 4.5 Hz, 1H), 2.40 (m, 2H), 2.20-2.07 (m, 3H), 2.01 (dtd, *J* = 17.0, 5.0, 2.5 Hz, 1H), 1.96-1.80 (m, 3H), 1.72 (m, 1H), 1.63-1.38 (m, 5H), 1.35-1.24 (m, 2H), 1.16-0.95 (m, 3H), 1.01 (s, 3H), 0.75 ppm (s, 3H); ^{13}C NMR (125 MHz, $CDCl_3$): d = 179.7, 141.0, 121.3, 80.3, 56.3, 55.6, 55.1, 50.1, 44.1, 38.6, 38.0, 37.2, 37.0, 32.0, 31.9, 27.9, 24.6, 23.4, 20.9, 19.4, 13.2 ppm; MS: *m/z* (%): 332 (22) (M^+), 300 (96), 285 (100), 261 (44), 258 (21), 161 (28), 145 (28), 105 (23), 91 (24).

Benzyl 2-acetylpyridinecarboxylate:[S11] a colorless oil: IR (neat): ν = 3125-2800, 1717, 1584, 1437, 1377, 1303, 1243, 1122, 1086, 1044, 995, 744, 697, 619 cm^{-1}; ^{1}H NMR (500 MHz, $CDCl_3$): δ = 8.77 (ddd, *J* = 5.0, 2.0, 1.0 Hz, 1H), 8.14 (dt, *J* = 8.0, 1.0 Hz, 1H), 7.83 (td, *J* = 8.0, 2.0 Hz, 1H), 7.51-7.46 (m, 3H), 7.41-7.32 (m, 3H), 5.46 ppm (s, 2H); ^{13}C NMR (125 MHz, $CDCl_3$): δ = 165.0, 150.0, 148.1, 137.0, 135.6, 128.6, 128.4 (3C), 127.0, 125.3, 67.6 ppm; MS: *m/z* (%): 213 (<1) (M^+), 168 (11), 107 (47), 91 (80), 79 (100), 65 (21), 51 (34).

***N*-(*tert*-butoxycarbonyl)-2,2-dimethyl-1,3-oxazolidine-4-carboxylic acid** (**34**):[S12] a colorless oil: IR (neat): ν = 3325-2775, 1694, 1368, 1169, 1096, 853 cm^{-1}; ^{1}H NMR (500 MHz, $CDCl_3$): δ = 4.52-4.39 (m, 1H), 4.35-4.10 (m, 2H), 1.73-1.63 (m, 2H), 1.51-1.39 ppm (m, 12H); MS: *m/z* (%): 230 (17) $[M\text{-}CH_3]^+$, 174 (18), 172 (17), 130 (93), 84 (29), 69 (23), 57 (100).

3. Spectra

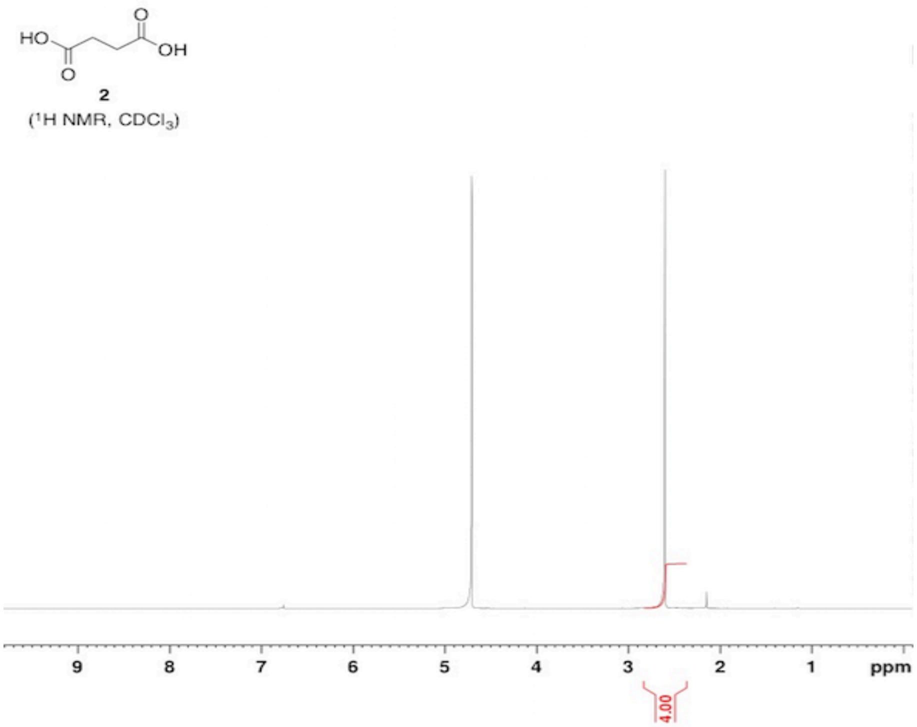

HO
OH
O
O
2
(1H NMR, CDCl3)
4.00
9
8
7
6
5
4
3
2
1
ppm

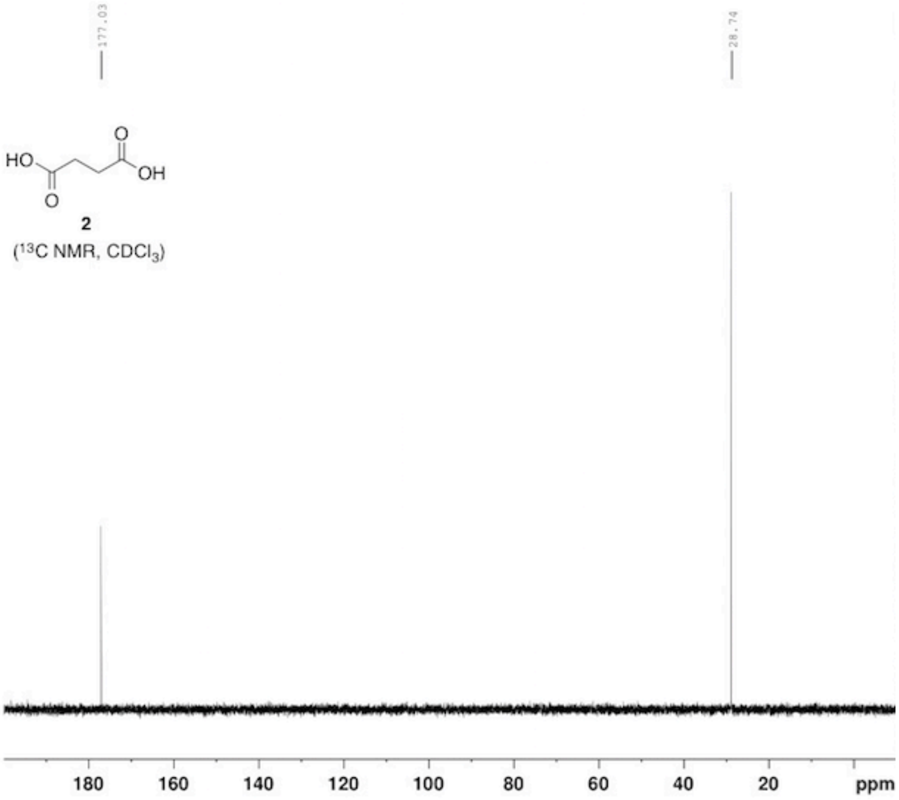

177.03
28.74
HO
OH
O
O
2
(13C NMR, CDCl3)
180
160
140
120
100
80
60
40
20
ppm

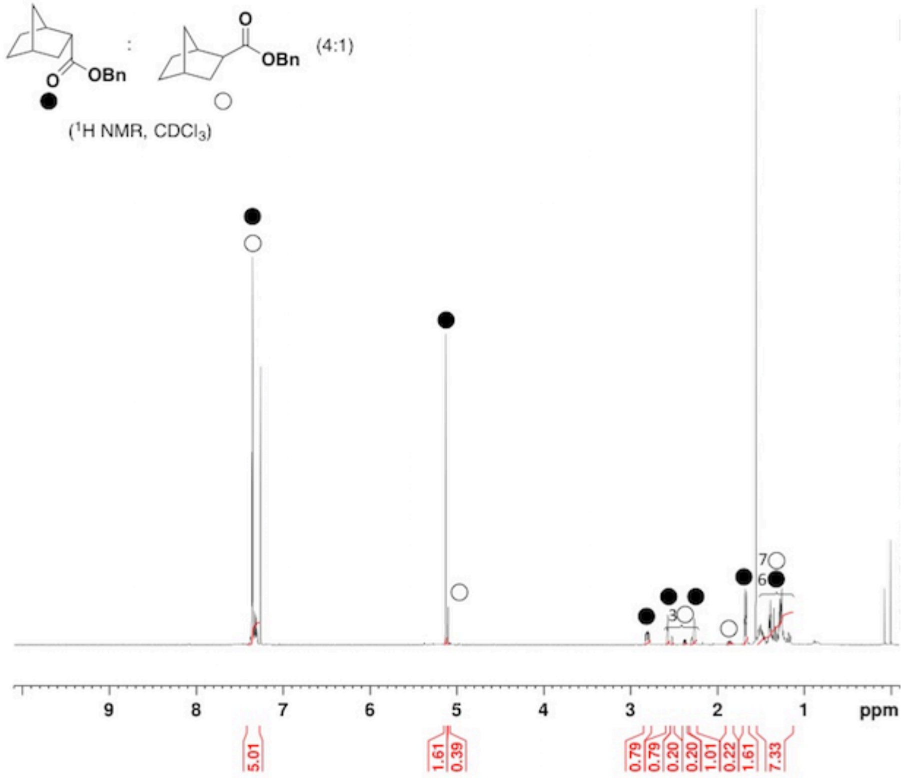
OBn
O
OBn
(4:1)
(1H NMR, CDCl3)
9 8 7 6 5 4 3 2 1 ppm
5.01
1.61
0.39
0.79
0.79
0.20
0.20
1.01
0.22
1.61
7.33

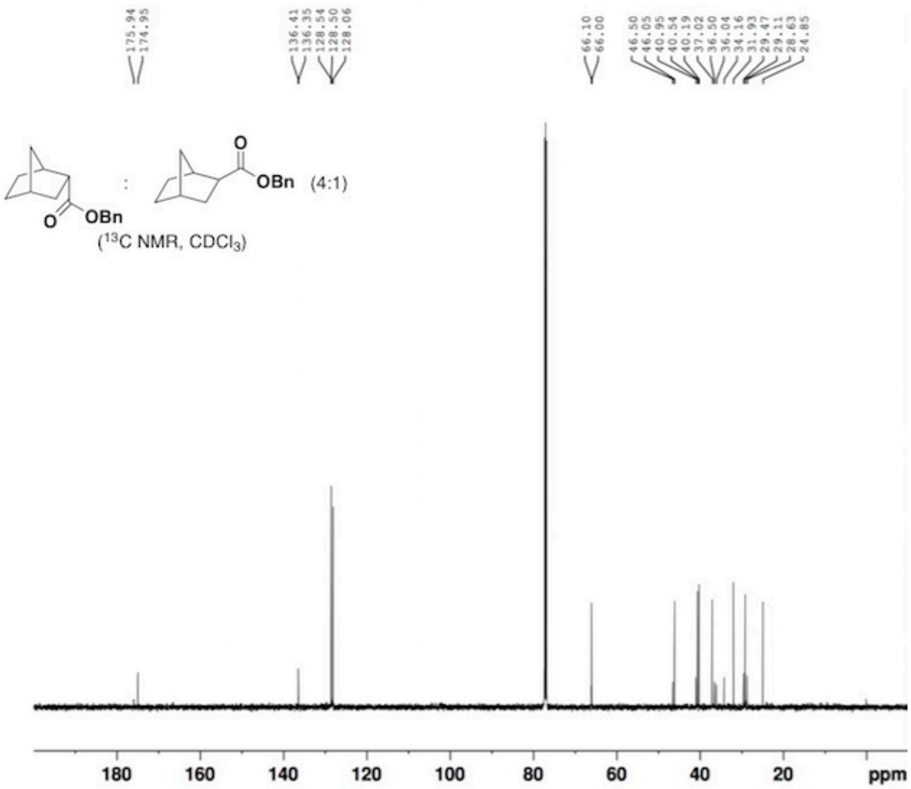
175.94
174.95
136.41
136.35
128.54
128.50
128.06
66.10
66.00
46.50
46.05
40.95
40.54
40.19
37.02
36.50
36.04
34.16
31.93
29.47
29.11
28.63
24.85
O
OBn
O
OBn
(4:1)
(13C NMR, CDCl3)
180 160 140 120 100 80 60 40 20 ppm

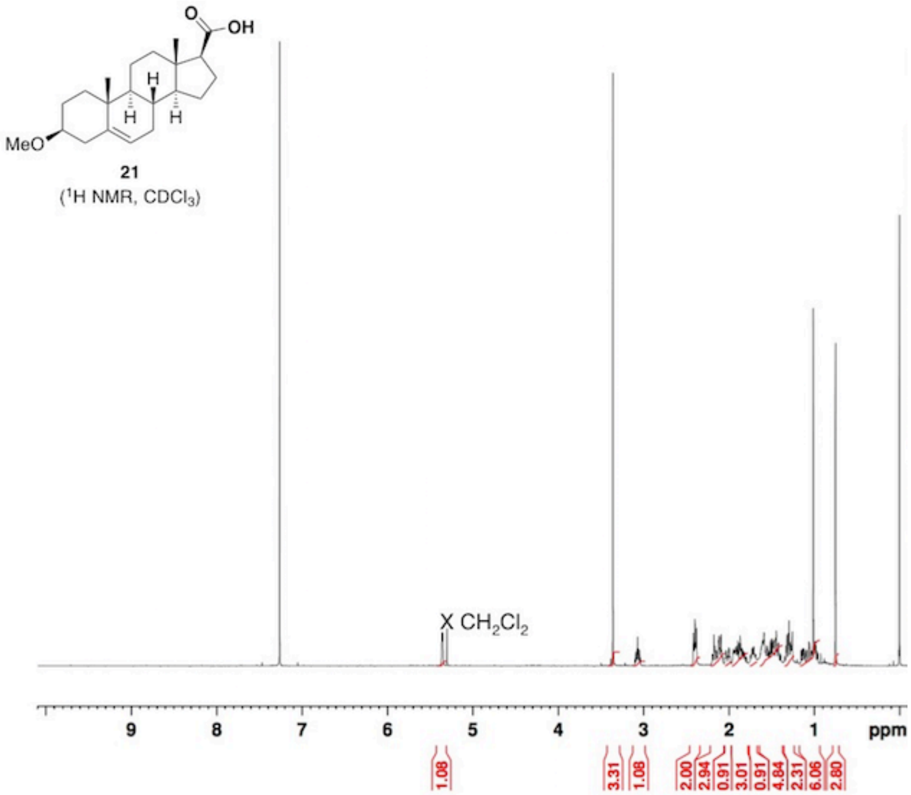
OH
H
MeO
21
(1H NMR, CDCl3)
X CH2Cl2
9 8 7 6 5 4 3 2 1 ppm
1.08
3.31
1.08
2.00
2.94
0.91
3.01
0.91
4.84
2.31
6.06
2.80

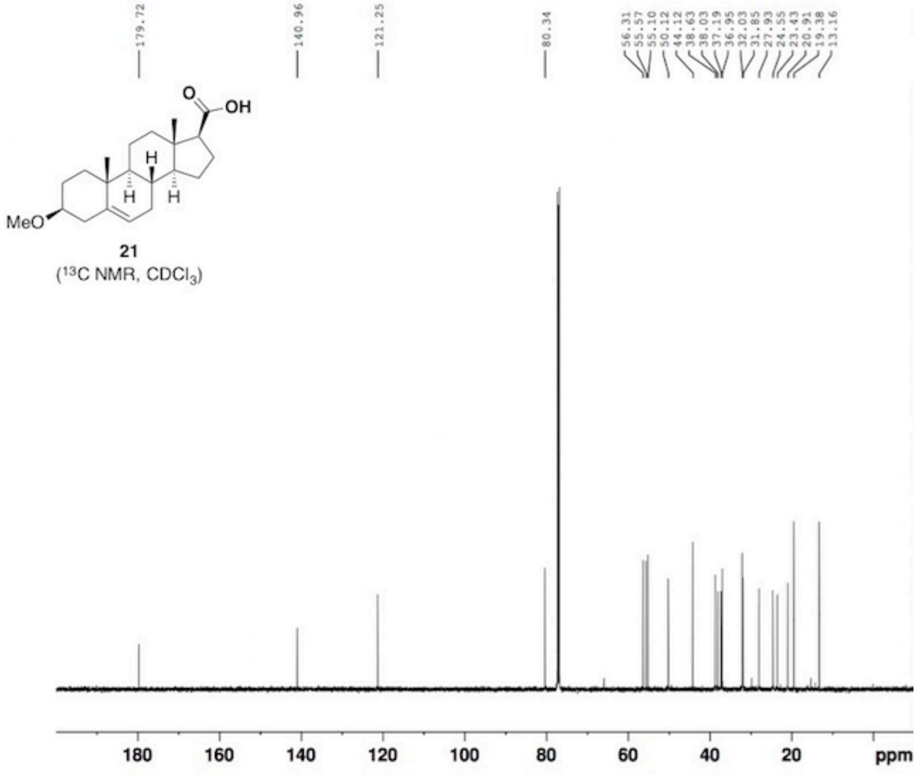
179.72
140.96
121.25
80.34
56.31
55.57
55.10
50.12
44.12
38.63
38.03
37.19
36.95
32.03
31.85
27.93
24.55
23.43
20.91
19.38
13.16
OH
H
MeO
21
(13C NMR, CDCl3)
180 160 140 120 100 80 60 40 20 ppm

4. Reference

S1 Nilsson, M., Duarte, I. F., Almeida, C., Delgadillo, I., Goodfellow, B. J., Gil, A. M., & Morris, G. A. *J. Agric. Food Chem.* **52**, 3736–3743 (2004).

S2 Grill, J. M., Ogle, J. W., Miller, S. A. *J. Org. Chem.* **71**, 9291–9296 (2006).

S3 Himeda, Y., Miyazawa, S., Onozawa-Komatsuzaki, N., Hirose, T., Kasuga, K. *Dalton Trans.* 6286–6288 (2009).

S4 den Hartog, T., Marciá, B., Minnaard, A. J., Feringa, B. L. *Adv. Synth. Catal.* **352**, 999–1013 (2010).

S5 Davidse, P. A., Dillen, J. L., Heyns, M. A. M., Modro, T. A., VanRooyen, P. H. *Can. J. Chem.* **68,** 741–746 (1990).

S6 Kobayashi, K., Kondo, Y. *Org. Lett.* **11**, 2035–2037 (2009).

S7 Yang, D., Yang, H., Fu, H. *Chem. Commun.* **47**, 2348–2350 (2011).

S8 Blaazer, A. R., Lange, J. H. M., van der Neut, M. A. W., Mulder, A., den Boon, F. S., Werkman, T. R., Kruse, C. G., Wadman, W. J. *Eur. J. Med. Chem.* **46**, 5086–5098 (2011).

S9 Kondo, T., Okada, T., Mitsudo, T. *Organometallics* **18**, 4123–4127 (1999).

S10 Romeu, A. M., Martino, E. E., Stoppani, A. O. M. *Biochim. Biophy. Acta, Lipids and Lipid Metabolism* **409**, 376–386 (1975).

S11 Frogneux, X., von Wolff, N., Thuery, P., Lefevre, G., Cantat, T. *Chem. Eur. J.* **22**, 2930-2934 (2016).

S12 Chattopadhyay, S. K., Biswas, S., Pal, B. K. *Synthesis* 1289–1294 (2006).
